# Supplementary material for: Preclinical Evaluation of the Systemic Safety, Efficacy, and Biodistribution of a Recombinant AAV8 Vector Expressing FIX-TripleL in Hemophilia B Mice: Implications for Human Gene Therapy
Source: Int J Mol Sci. 2025 Jun 24;26(13):6073. doi: 10.3390/ijms26136073 (PMC12250290; doi:10.3390/ijms26136073)
Supplement: Supplementary file 1 [file ijms-26-06073-s001.zip › supplementary data.pdf]

Supplementary data

Supplementary Figure S1 FIX activity following intravenous administration of AAV8-FIX-TripleL in hemophilia B mice at subtherapeutic and long-term therapeutic doses

Supplementary Figure S2 Body weights of mice at various time points following AAV8-FIX-TripleL administration.

Supplementary Figure S3 Subcutaneous hemorrhage in hemophilia B mice and its improvement following gene therapy

Supplementary Figure S4 Representative images of histopathologic findings

Supplementary Table S1 Detection rates of the biodistribution of AAV8-FIX-TripleL following intravenous injection in hemophilia B mice (n = 15 per group)

Supplementary Table S2 Quality test items and results for AAV8-FIX-TripleL

Supplementary Table S3 Comprehensive hematology parameters on Day 2

Supplementary Table S4 Comprehensive hematology parameters on Day 15

Supplementary Table S5 Comprehensive hematology parameters on Day 29

Supplementary Table S6 Comprehensive hematology parameters on Day 91

Supplementary Table S7 Comprehensive biochemistry parameters on day 2

Supplementary Table S8 Comprehensive biochemistry parameters on day 15

Supplementary Table S9 Comprehensive biochemistry parameters on day 29

Supplementary Table S10 Comprehensive biochemistry parameters on day 91.

Supplementary Table S11 Comprehensive urine quantitative analysis parameters on day 2.

Supplementary Table S12 Comprehensive urine quantitative analysis parameters on Day 15

Supplementary Table S13 Comprehensive urine quantitative analysis parameters on Day 29

Supplementary Table S14 Comprehensive urine quantitative analysis parameters on Day 91

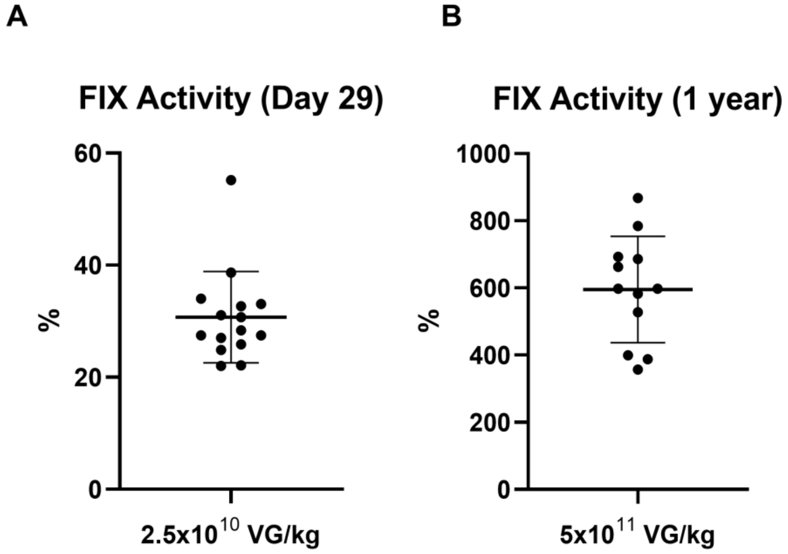

Supplementary Figure S1.

FIX activity following intravenous administration of AAV8-FIX-TripleL in hemophilia B mice at subtherapeutic and long-term therapeutic doses

Circulating human FIX activity was measured using a one-stage clotting assay (FIX-specific activated partial thromboplastin time). This study was conducted as a non-GLP experiment. Hemophilia B mice were injected intravenously with AAV8-FIX-TripleL, and plasma samples were collected to assess FIX activity. Data are shown as dot plots with means ± SD.

(A) FIX activity at a subtherapeutic dose of 2.5×10^{10} VG/kg, with mice sacrificed on Day 29 (n = 15 per group).

(B) FIX activity at a therapeutic dose of 5×10^{11} VG/kg, with mice sacrificed at 1 year post-injection (n = 12 per group).

hFIX, human factor IX; FIX, factor IX; AAV, adeno-associated virus; SD, standard deviation; VG, viral genome.

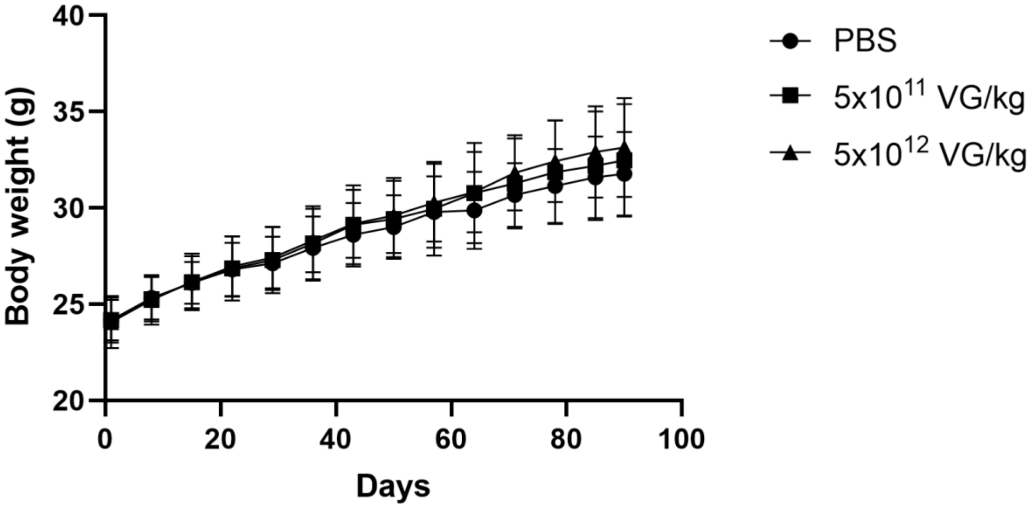

Supplementary Figure S2

Body weights of mice at various time points following AAV8-FIX-TripleL administration.

FIX, factor IX; AAV, adeno-associated virus; VG, viral genome.

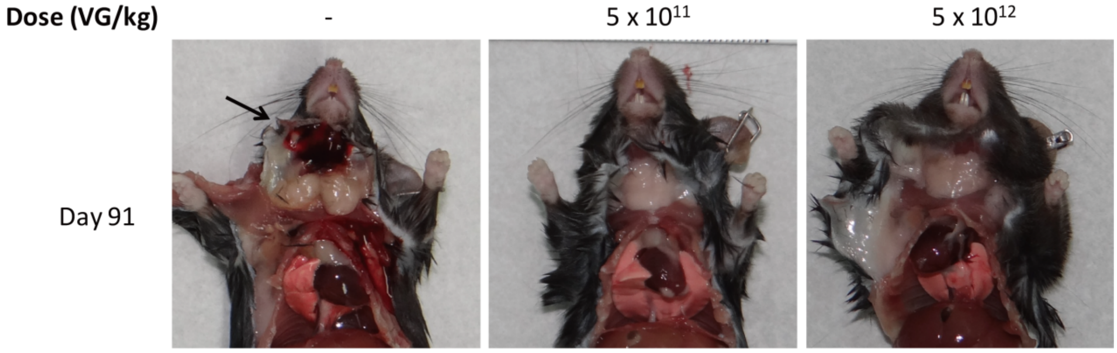

Supplementary Figure S3.

Subcutaneous hemorrhage in hemophilia B mice and its improvement following gene therapy

Representative gross necropsy images taken on Day 91 showing subcutaneous hemorrhage in hemophilia B (HB) mice and the therapeutic effect of AAV8-FIX-TripleL gene therapy. From left to right: control group, therapeutic dose group (5×10^{11} VG/kg), and supraphysiological dose group (5×10^{12} VG/kg).

FIX, factor IX; AAV, adeno-associated virus; VG, viral genome.

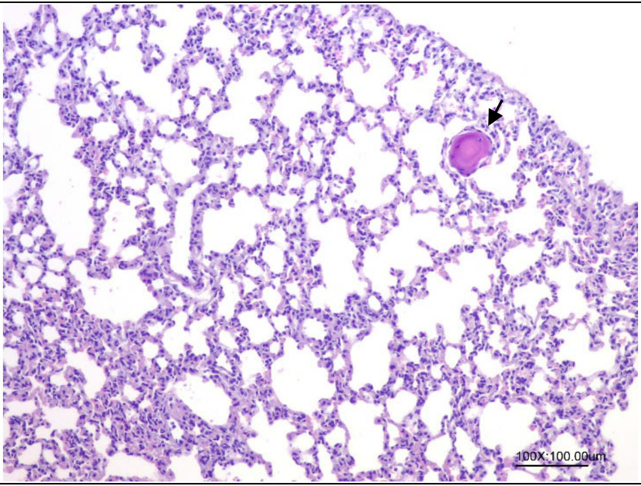

Sup. Fig. S4-1. Sample No.:MI052 (100X, H&E)
Control group, Day 2, osseous metaplasia, alveoli, focal, minimal, lung.

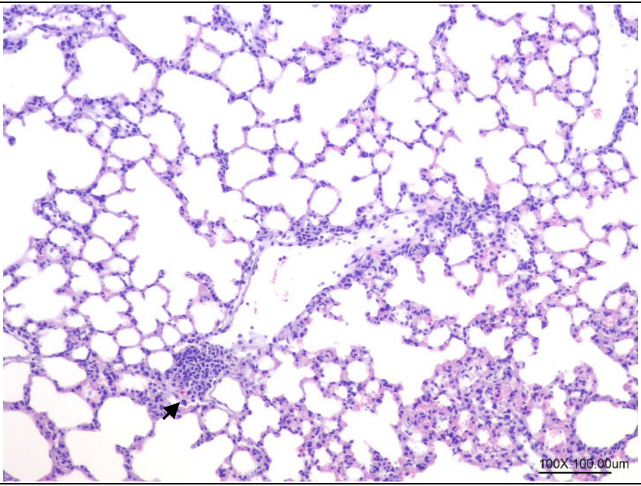

Sup. Fig. S4-2. Sample No.: MI053 (100X, H&E)
Supraphysiological dose group, Day 2, infiltration, mononuclear cell, focal, minimal, lung.

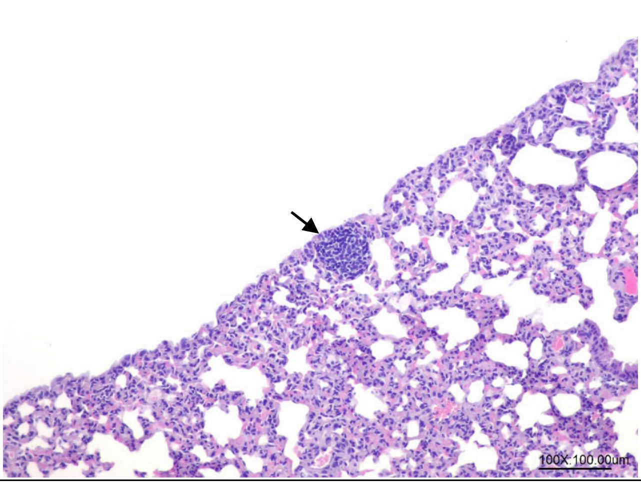

Sup. Fig. S4-3. Sample No.: MI159 (100X, H&E)
Supraphysiological dose group, Day 15, infiltration, mononuclear cell, focal, minimal, lung.

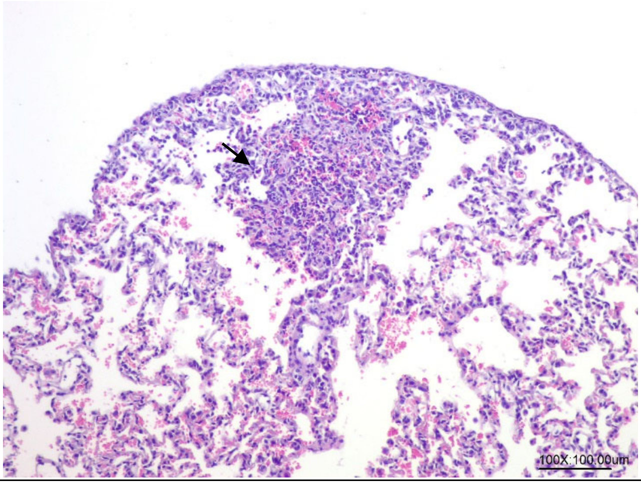

Sup. Fig. S4-4. Sample No.: MI098 (100X, H&E)
Control group, Day 91, infiltration, mononuclear cell, focal, minimal, lung.

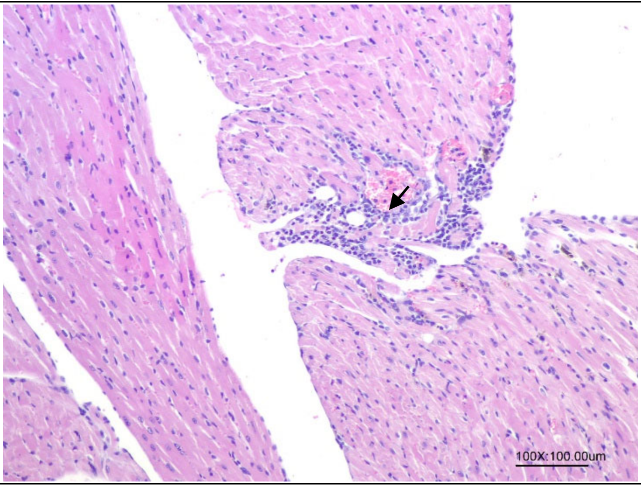

Sup. Fig. S4-5. Sample No.: MI343 (100X, H&E)
Control group, Day 2, infiltration, mononuclear cell, focal, minimal, lung.

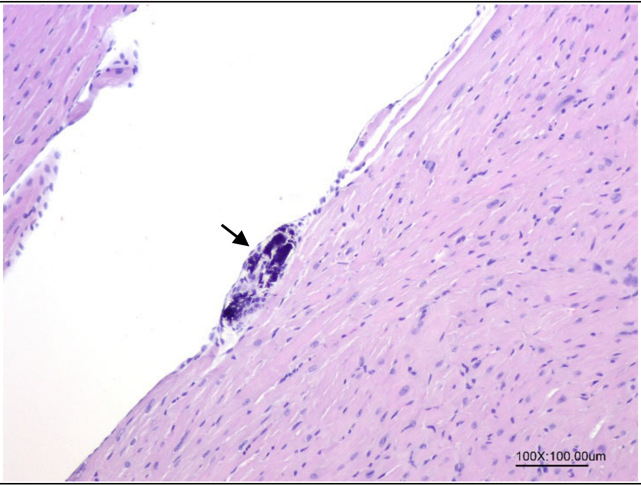

Sup. Fig. S4-6. Sample No.: MI181 (100X, H&E)
Control group, Day 15, mineralization, focal, minimal, heart.

(Continued)

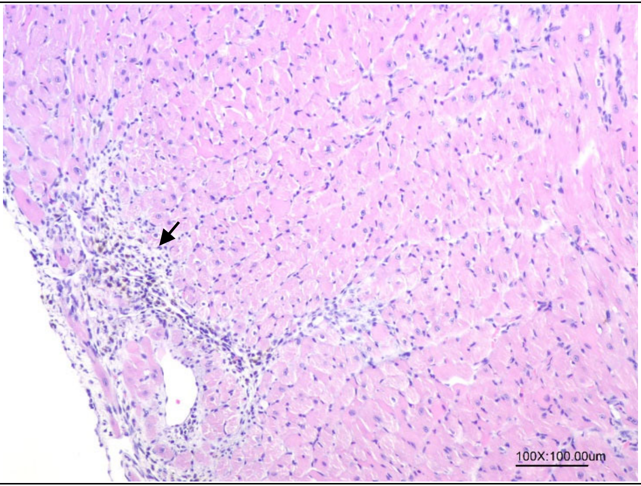

Sup. Fig. S4-7. Sample No.: MI215 (100X, H&E) Supraphysiological dose group, Day 29, cardiomyopathy, focal, minimal, heart.

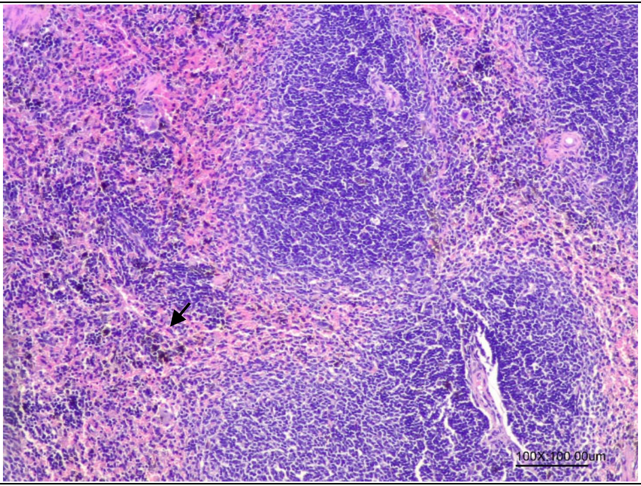

Sup. Fig. S4-8. Sample No.: MI357 (100X, H&E) Control group, Day 2, pigment, focal, minimal, spleen.

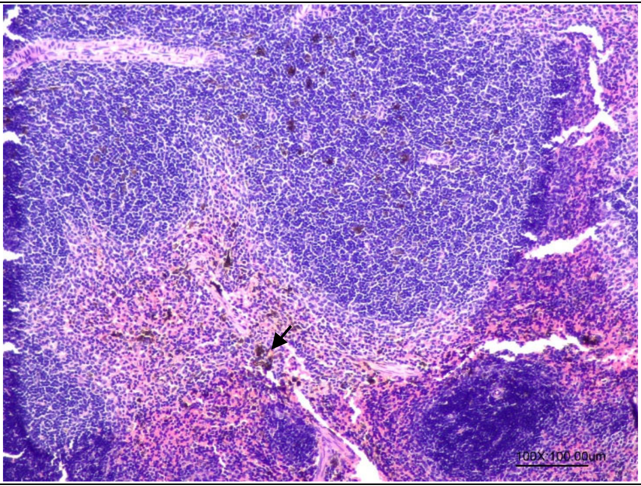

Sup. Fig. S4-9. Sample No.: MI375 (100X, H&E) Therapeutic dose group, Day 2, pigment, focal, minimal, spleen.

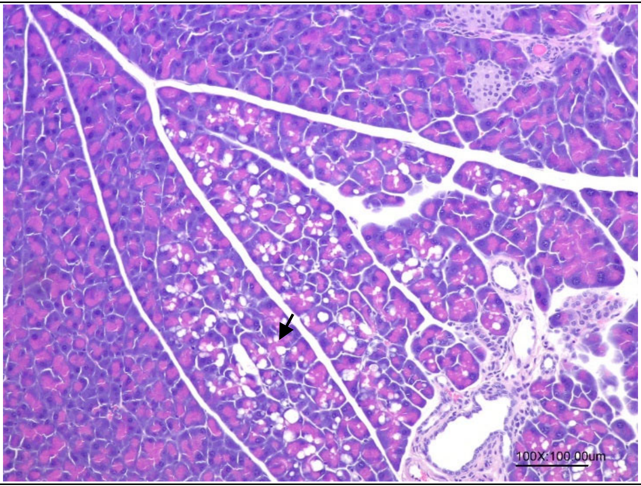

Sup. Fig. S4-10. Sample No.:MI065 (100X, H&E) Control group, Day 2, vacuolation, focal, minimal, pancreas.

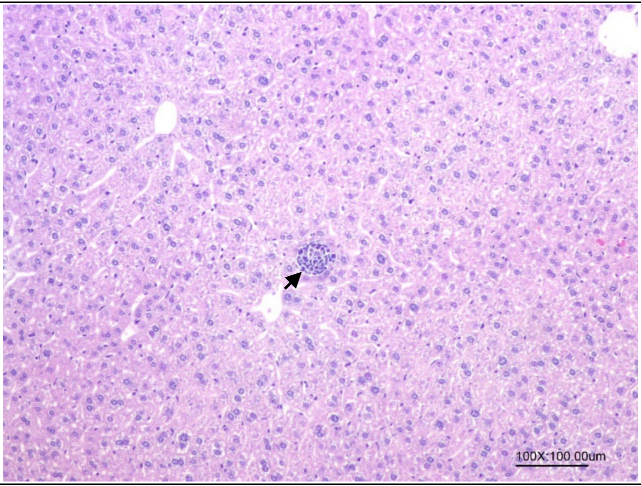

Sup. Fig. S4-11. Sample No.: MI052 (100X, H&E) Control group, Day 2, infiltration, mononuclear cell, focal, minimal, liver.

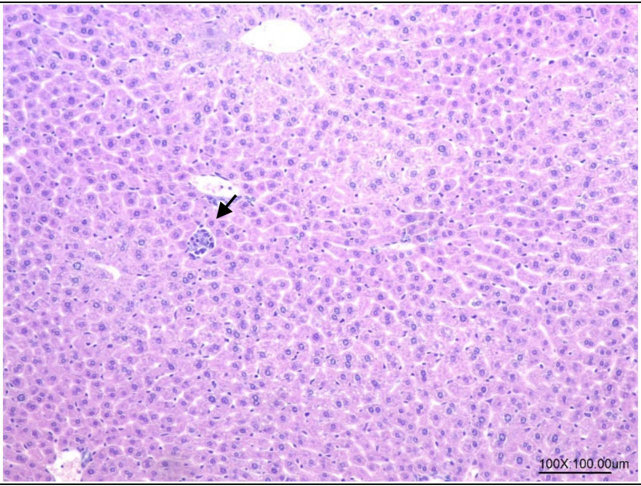

Sup. Fig. S4-12. Sample No.: MI006 (100X, H&E) Therapeutic dose group, Day 2, infiltration, mononuclear cell, focal, minimal, liver.

(Continued)

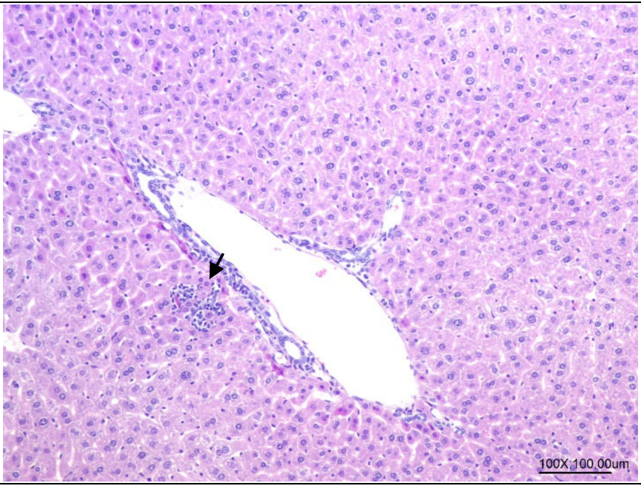

Sup. Fig. S4-13. Sample No.:MI022 (100X, H&E) Supraphysiological dose group, Day 2, infiltration, mononuclear cell, focal, minimal, liver.

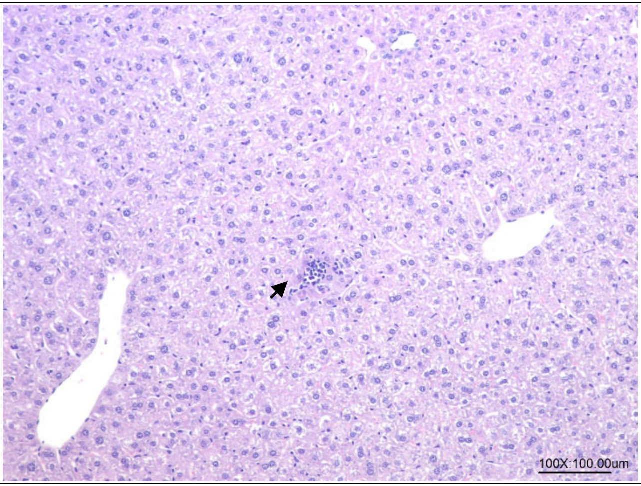

Sup. Fig. S4-14. Sample No.: MI144 (100X, H&E) Control group, Day 15, infiltration, mononuclear cell, focal, minimal, liver.

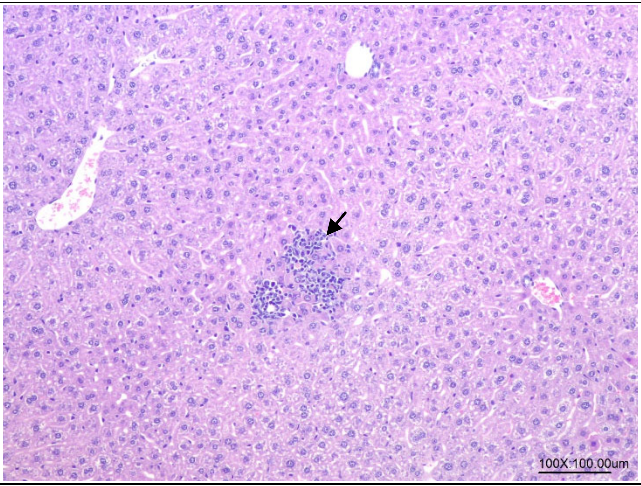

Sup. Fig. S4-15. Sample No.: MI153 (100X, H&E) Therapeutic dose group, Day 15, infiltration, mononuclear cell, focal, minimal, liver.

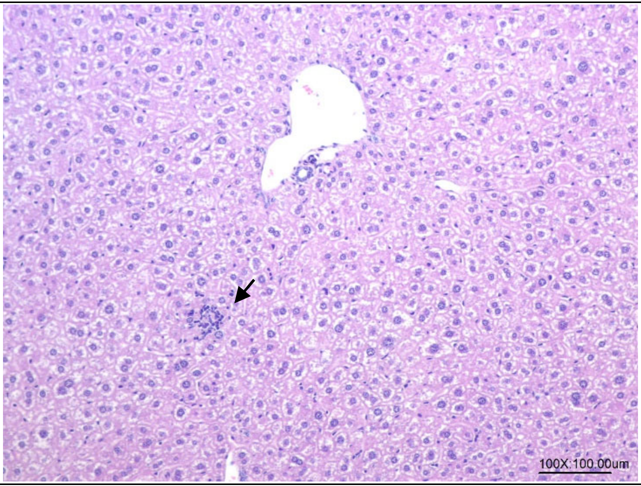

Sup. Fig. S4-16. Sample No.: MI135 (100X, H&E) Control group, Day 91, infiltration, mononuclear cell, focal, minimal, liver.

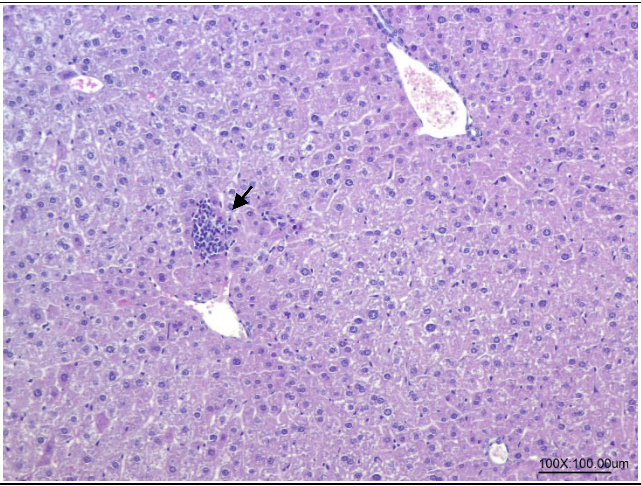

Sup. Fig. S4-17. Sample No.: MI100 (100X, H&E) Therapeutic dose group, Day 91, infiltration, mononuclear cell, focal, minimal, liver.

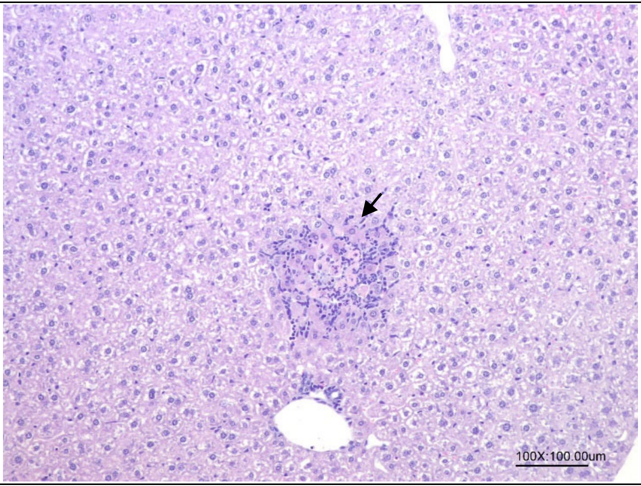

Sup. Fig. S4-18. Sample No.: MI173 (100X, H&E) Control group, Day 15, necrosis, focal, minimal, liver.

(Continued)

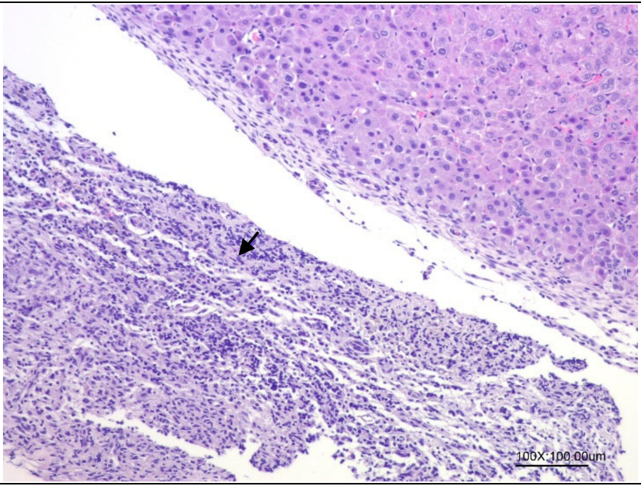

Sup. Fig. S4-19. Sample No.: MI215 (100X, H&E) Supraphysiological dose group, Day 29, fibrosis, focal, mild, liver.

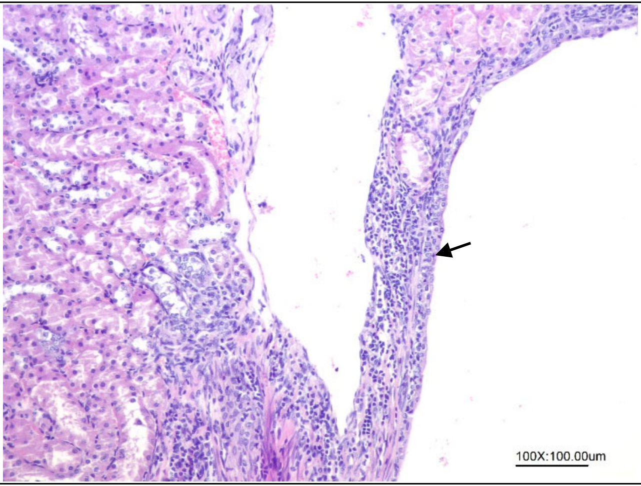

Sup. Fig. S4-20. Sample No.: MI024 (100X, H&E) Control group, Day 2, infiltration, mononuclear cell, cortex, focal, minimal, kidneys.

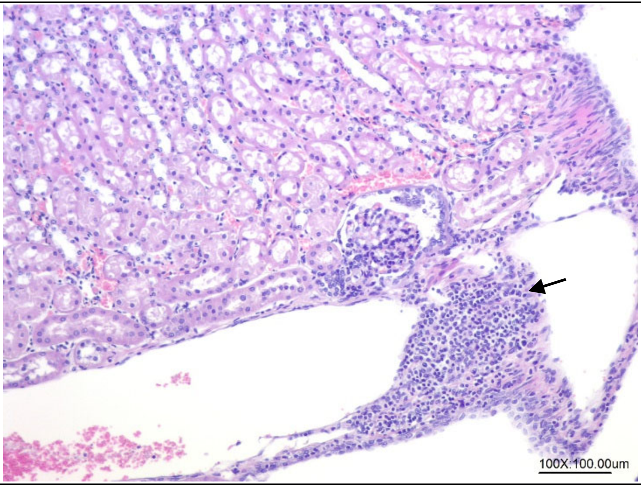

Sup. Fig. S4-21. Sample No.:MI375 (100X, H&E) Therapeutic dose group, Day 2, infiltration, mononuclear cell, cortex, focal, minimal, kidneys.

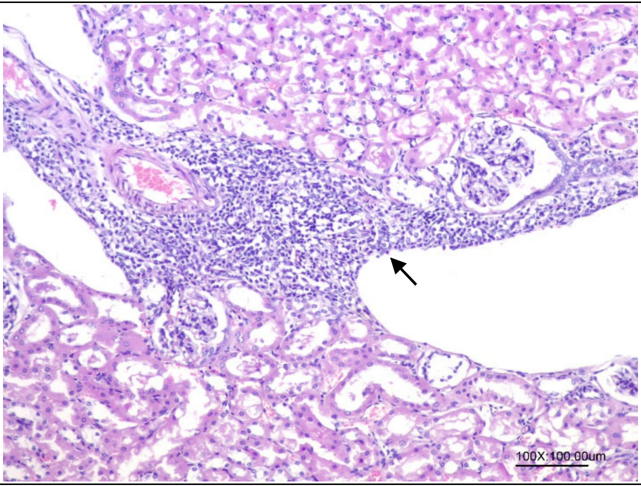

Sup. Fig. S4-22. Sample No.:MI362 (100X, H&E) Supraphysiological dose group, Day 2, infiltration, mononuclear cell, cortex, focal, minimal, kidneys.

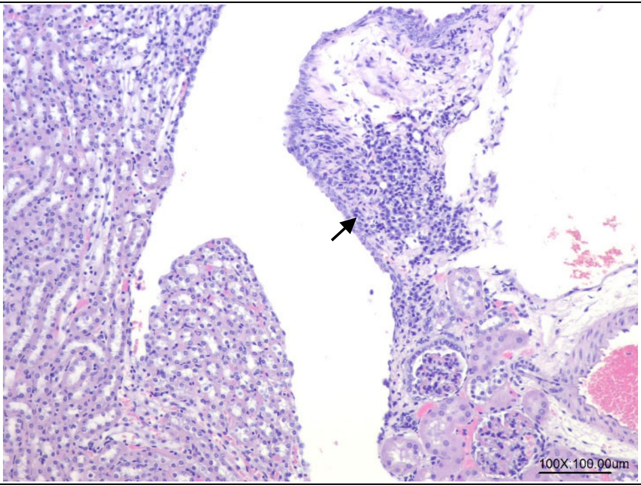

Sup. Fig. S4-23. Sample No.:MI178 (100X, H&E) Control group, Day 15, infiltration, mononuclear cell, cortex, focal, minimal, kidneys.

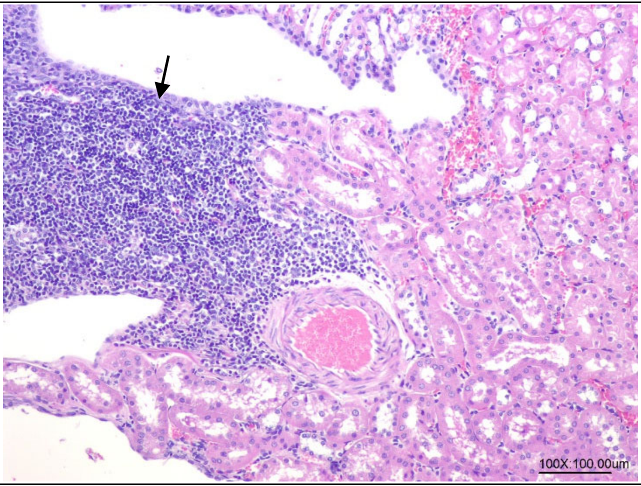

Sup. Fig. S4-24. Sample No.:MI141 (100X, H&E) Supraphysiological dose group, Day 91, infiltration, mononuclear cell, cortex, focal, minimal, kidneys.

(Continued)

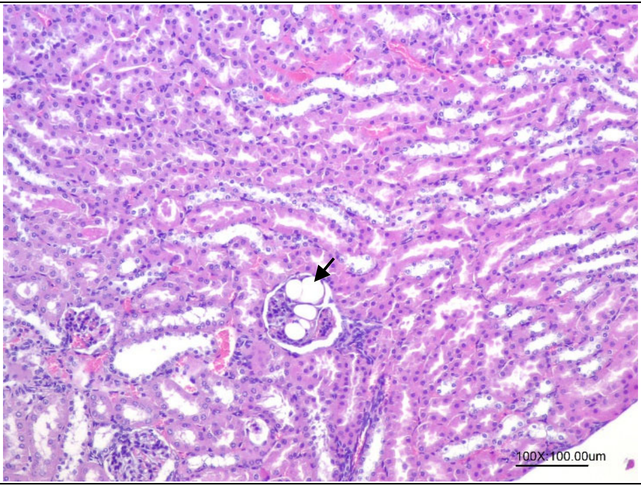

Sup. Fig. S4-25. Sample No.: MI007 (100X, H&E) Supraphysiological dose group, Day 2, vacuolation, glomerulus, focal, minimal, kidneys.

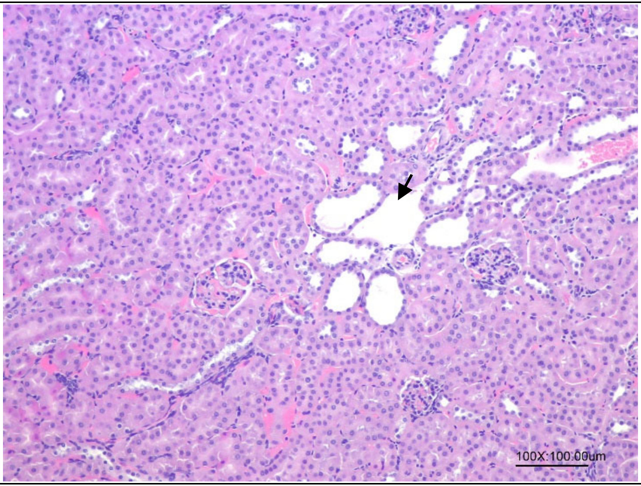

Sup. Fig. S4-26. Sample No.:MI231 (100X, H&E) Control group, Day 29, dilation, tubule, cortex, focal, minimal, kidneys.

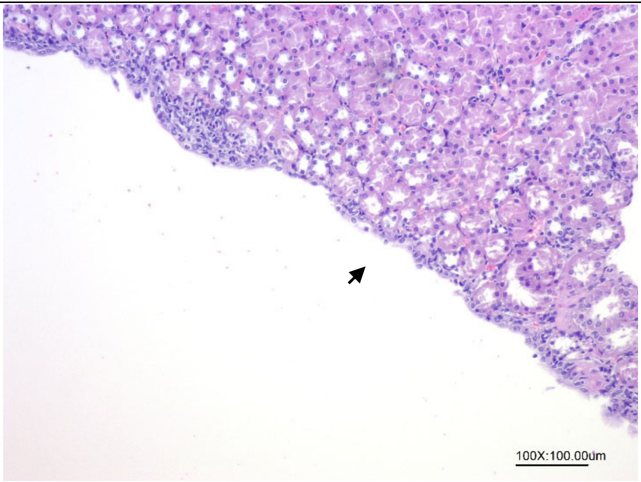

Sup. Fig. S4-27. Sample No.: MI024 (100X, H&E) Control group, Day 2, cyst, medulla, focal, moderate, kidneys.

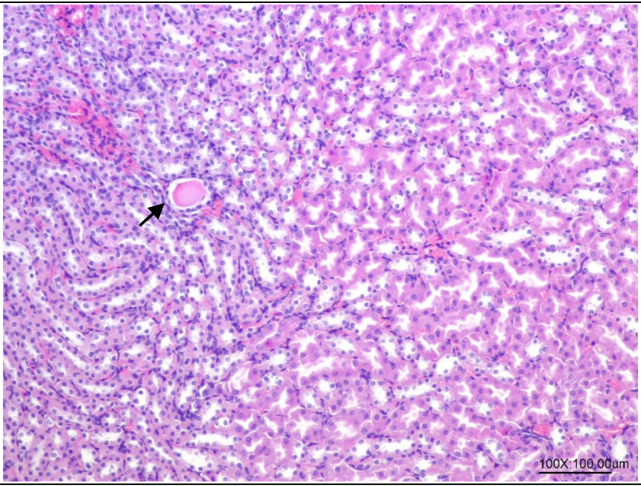

Sup. Fig. S4-28. Sample No.:MI065 (100X, H&E) Control group, Day 2, cast, renal tubule, cortex, focal, minimal, kidneys.

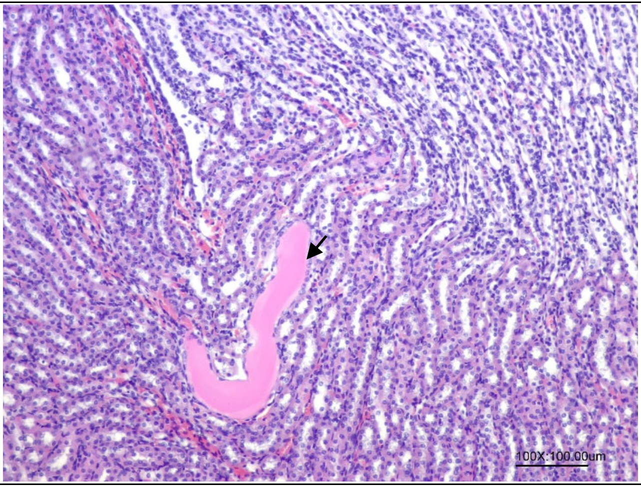

Sup. Fig. S4-29. Sample No.: MI152 (100X, H&E) Therapeutic dose group, Day 15, cast, renal tubule, cortex, focal, minimal, kidneys.

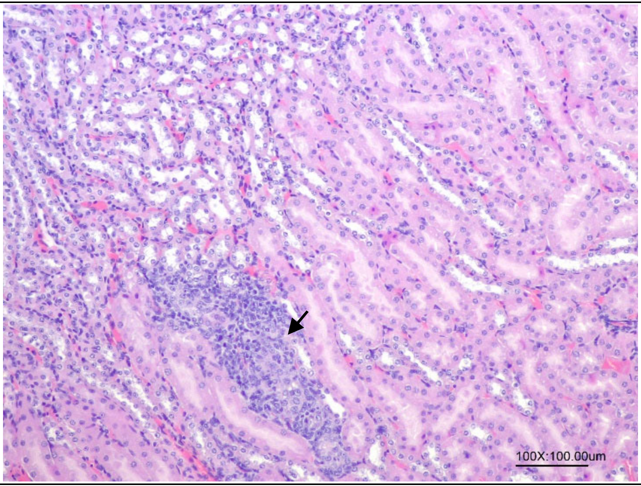

Sup. Fig. S4-30. Sample No.: MI158 (100X, H&E) Therapeutic dose group, Day 15, basophilia, tubule, cortex, focal, minimal, kidneys.

(Continued)

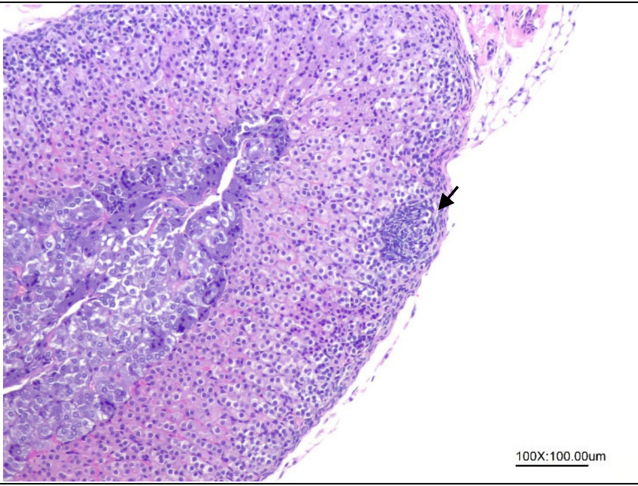

Sup. Fig. S4-31. Sample No.: MI357 (100X, H&E) Control group, Day 2, hyperplasia, subcapsular, cortex, focal, minimal, adrenals.

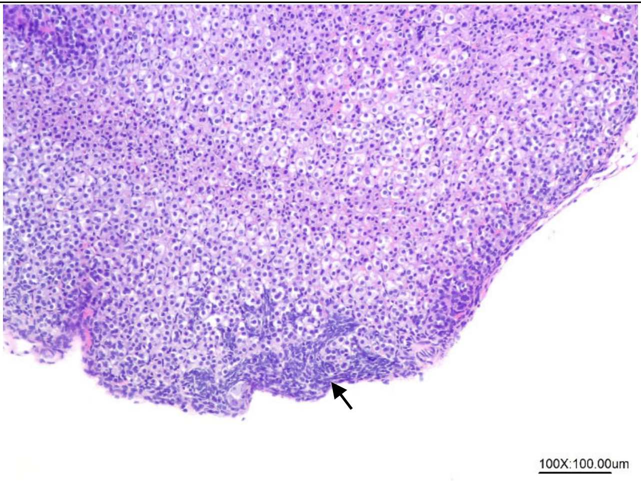

Sup. Fig. S4-32. Sample No.:MI078 (100X, H&E) Supraphysiological dose group, Day 2, hyperplasia, subcapsular, cortex, focal, minimal, adrenals.

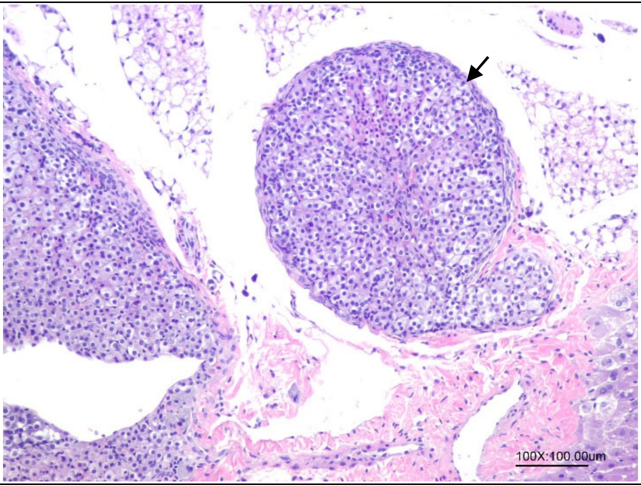

Sup. Fig. S4-33. Sample No.: MI925 (100X, H&E) Therapeutic dose group, Day 2, accessory adrenocortical nodule, focal, mild, adrenals.

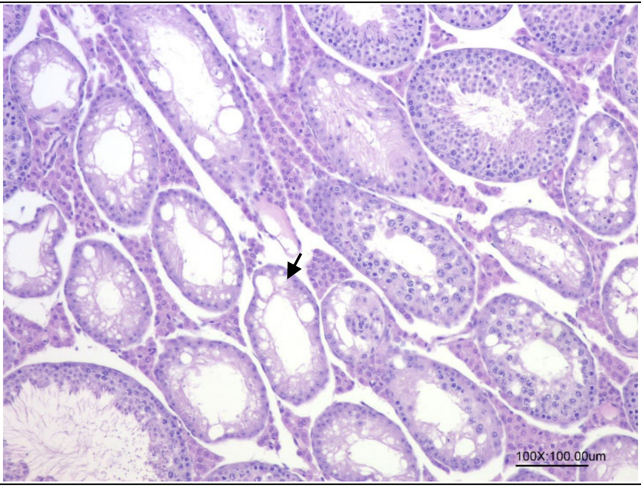

Sup. Fig. S4-34. Sample No.:MI199 (100X, H&E) Therapeutic dose group, Day 29, degeneration, germ cell, focal, moderate, testis.

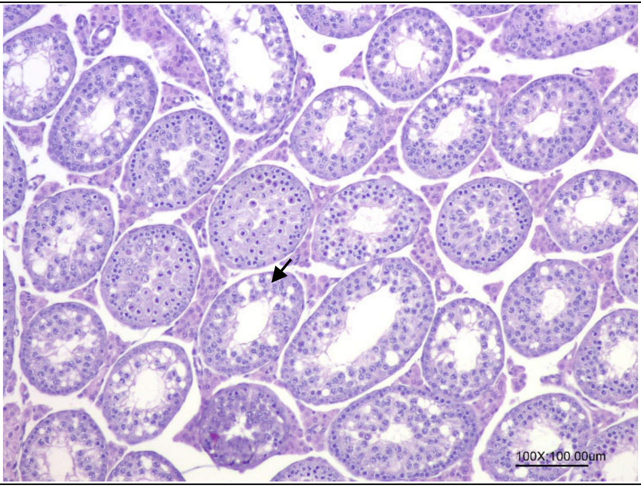

Sup. Fig. S4-35. Sample No.: MI202 (100X, H&E) Control group, Day 29, degeneration, germ cell, focal, moderate, testis.

(Continued)

Supplementary Figure S4

Representative images of histopathologic findings

Supplementary Figures 4-1 to 4-35 present representative histopathologic images obtained from this study, stained with hematoxylin and eosin (H&E) at 100× magnification. Key findings are indicated by arrows in each figure.

Supplementary Table S1 Detection rates of the biodistribution of AAV8-FIX-TripleL following intravenous injection in hemophilia B mice (n = 15 per group)

AAV8-FIX-TripleL DNA/total no. of animals												
**Days**	Day 2			Day 15			Day 29			Day 91		
**Dose (VG/kg)**	-	$5x10^{11}$	$5x10^{12}$	-	$5x10^{11}$	$5x10^{12}$	-	$5x10^{11}$	$5x10^{12}$	-	$5x10^{11}$	$5x10^{12}$
**Heart**	0/15	2/15	15/15	0/15	0/15	15/15	0/15	0/15	13/15	0/15	0/15	9/15
**Liver**	0/15	15/15	15/15	0/15	15/15	15/15	0/15	15/15	15/15	0/15	15/15	15/15
**Spleen**	0/15	8/15	15/15	0/15	2/15	15/15	0/15	0/15	5/15	0/15	0/15	0/15
**Lung**	0/15	0/15	15/15	0/15	0/15	6/15	0/15	0/15	3/15	0/15	0/15	0/15
**Kidney**	0/15	1/15	15/15	0/15	0/15	12/15	0/15	0/15	12/15	0/15	0/15	0/15
**Brain**	0/15	1/15	8/15	0/15	0/15	2/15	0/15	0/15	2/15	0/15	0/15	1/15
**Testis**	0/15	0/15	15/15	0/15	0/15	15/15	0/15	0/15	4/15	0/15	0/15	0/15

FIX, factor IX; AAV, adeno-associated virus; VG, viral genome.

Supplementary Table S2 Quality test items and results of AAV8-FIX-TripleL

**Test Items**			**Acceptance Criteria**	**Results**
General	Appearance		Clear, free of foreign materials	Clear solution, free of foreign materials
	pH		pH 6.5-8	7.33
	Osmolality		250-400 mOsm/kg (250-400 mOsm)	307 mOsm/kg
Identity	AAV protein content		VP1,2,3 present	VP1,2,3 present
Purity	AAV purity (SDS–PAGE)		≥ 80% capsid purity	85.71%
	(Residual) Host cell proteins		Record results	<LLOQ (LLOQ: 10 ng/mL)
	(Residual) Host cell DNA		Record results	Undetectable (LOD: 19.2 ng/mL)
	(Residual) Plasmid DNA (Amp^{R})		≤100 ng/mL	15.9 ng/mL ($6.27 x 10^{9}$ copies/mL)
	Empty:full particle ratio (TEM)		Record results	69.20%
Quality	Viral genome titer	ITR	≥ 1E+12 VG/mL	6.03×10^{13} VG/mL
		FIX-TripleL		2.71×10^{12} VG/mL
	AAV capsid titer		Record results	2.97×10^{13} capsids/mL
	Infectivity titer		Record results	3.98×10^{7} IU/mL
Safety	Endotoxin		≤ 5EU/mL	<0.1 EU/mL
	Sterility		Negative	Negative
	Mycoplasma (PCR-based)		Negative	Negative

AAV, adeno-associated virus; VP, viral protein; LLOQ, lower limit of qualification; LOD, limit of detection; VG, viral genome; IU, infectious units; EU, endotoxin units.

Supplementary Table S3 Comprehensive hematology parameters on Day 2

Parameters	Dose (VG/kg)		
	-	5 x 10^{11}	5 x 10^{12}
White Blood Cells, WBCs (10^3/μL)	5.28±1.97	6.30±1.79	5.92±2.17
Red Blood Cells, RBCs (10^6/μL)	9.42±0.62	9.13±0.60	9.28±0.47
Hemoglobin, HGB (g/dL)	13.54±0.84	13.07±0.85	13.17±0.66
Hematocrit, HCT (%)	42.60±1.85	**40.87±1.85***	41.41±2.04
Mean Corpuscular Volume, MCV (fL)	45.35±2.58	44.85±1.52	44.71±2.47
Mean Corpuscular Hemoglobin, MCH (pg)	14.39±0.53	14.33±0.26	14.21±0.37
Mean Corpuscular Hemoglobin Concentration, MCHC (g/dL)	31.76±0.88	31.95±0.87	31.83±1.02
Platelet, PLT (10^3/μL)	1636±229	1679±181	1737±208
Red Cell Distribution Width, RDW-SD (fL)	31.31±6.20	28.78±3.06	28.85±4.60
Red Cell Distribution Width, RDW-CV (%)	21.73±2.01	20.75±1.57	20.78±1.45
Platelet Distribution Width, PDW (fL)	6.79±0.32	6.63±0.14	6.83±0.46
Mean Platelet Volume, MPV (fL)	6.35±0.26	6.30±0.13	6.41±0.27
Platelet Large Cell Ratio, P-LCR (%)	3.51±1.08	3.19±0.43	3.68±1.20
Plateletcrit, PCT (%)	1.04±0.13	1.06±0.12	1.11±0.12
Neutrophils, NEUT (10^3/μL)	0.50±0.25	0.53±0.17	0.54±0.20
Lymphocytes, LYMPH (10^3/μL)	4.58±1.80	5.53±1.63	5.15±2.01
Monocytes, MONO (10^3/μL)	0.16±0.08	0.20±0.08	0.18±0.09
Eosinophils, EOS (10^3/μL)	0.04±0.03	0.04±0.02	0.05±0.04
Basophils, BASO (10^3/μL)	0.000±0.000	0.000±0.000	0.001±0.003
NEUT (%)	10.3±5.1	8.7±3.0	10.3±5.4
LYMPH (%)	86.2±5.1	87.4±3.3	85.9±4.5
MONO (%)	2.8±0.8	3.2±0.8	3.0±0.5
EOS (%)	0.72±0.57	0.67±0.35	0.8±0.6
BASO (%)	0.00±0.00	0.00±0.00	0.01±0.03
Reticulocytes, RET (10^6/μL)	0.5188±0.1752	0.5439±0.1940	0.5857±0.6072
RET (%)	5.61±2.26	6.07±2.56	6.49±7.31

*: $p < 0.05$ (compared with the vehicle group; unpaired Student's t test).

Historical control data

HCT (%): 39.56±3.30

VG, viral genome.

Supplementary Table S4 Comprehensive hematology parameters on Day 15

Parameters	Dose (VG/kg)		
	-	5 x 10^{11}	5 x 10^{12}
White Blood Cells, WBCs (10^3/μL)	6.15±1.91	5.32±2.00	5.76±2.02
Red Blood Cells, RBCs (10^6/μL)	9.67±0.28	9.54±0.37	9.75±0.21
Hemoglobin, HGB (g/dL)	13.69±0.50	13.49±0.39	13.77±0.29
Hematocrit, HCT (%)	42.57±1.80	42.27±1.16	42.77±1.02
Mean Corpuscular Volume, MCV (fL)	44.02±1.37	44.36±1.43	43.86±0.85
Mean Corpuscular Hemoglobin, MCH (pg)	14.15±0.31	14.16±0.27	14.12±0.16
Mean Corpuscular Hemoglobin Concentration, MCHC (g/dL)	32.18±0.66	31.92±0.67	32.20±0.40
Platelet, PLT (10^3/μL)	1726±271	1679±163	1724±166
Red Cell Distribution Width, RDW-SD (fL)	27.83±2.64	27.79±2.25	27.97±1.48
Red Cell Distribution Width, RDW-CV (%)	20.58±0.89	20.47±0.89	20.83±0.83
Platelet Distribution Width, PDW (fL)	6.69±0.24	6.64±0.21	6.59±0.16
Mean Platelet Volume, MPV (fL)	6.29±0.18	6.29±0.17	6.23±0.06
Platelet Large Cell Ratio, P-LCR (%)	3.21±0.58	3.33±0.73	3.01±0.30
Plateletcrit, PCT (%)	1.09±0.16	1.06±0.10	1.07±0.11
Neutrophils, NEUT (10^3/μL)	0.50±0.21	0.41±0.18	0.47±0.21
Lymphocytes, LYMPH (10^3/μL)	5.39±1.67	4.67±1.75	5.01±1.81
Monocytes, MONO (10^3/μL)	0.20±0.09	0.19±0.08	0.21±0.07
Eosinophils, EOS (10^3/μL)	0.06±0.05	0.05±0.04	0.06±0.02
Basophils, BASO (10^3/μL)	0.003±0.005	**0.000±0.000***	**0.000±0.000***
NEUT (%)	8.3±2.6	7.7±1.3	8.3±2.4
LYMPH (%)	87.5±2.7	88.0±1.8	86.8±2.6
MONO (%)	3.2±0.7	3.5±0.6	3.8±0.9
EOS (%)	0.93±0.61	0.93±0.81	1.11±0.36
BASO (%)	0.05±0.07	**0.00±0.00***	**0.00±0.00***
Reticulocytes, RET (10^6/μL)	0.3899±0.0502	0.3697±0.0355	0.3679±0.0483
RET (%)	4.03±0.49	3.88±0.40	3.77±0.51

*: $p < 0.05$ (compared with the vehicle group; unpaired Student's t test).

Historical control data

BASO (10^3/μL): <0.001

BASO (%): <0.01

VG, viral genome.

Supplementary Table S5 Comprehensive hematology parameters on Day 29

Parameters	Dose (VG/kg)		
	-	5 x 10^{11}	5 x 10^{12}
White Blood Cells, WBCs (10^3/μL)	6.55±1.75	6.22±1.37	6.02±2.04
Red Blood Cells, RBCs (10^6/μL)	9.66±0.38	9.72±0.23	9.66±0.38
Hemoglobin, HGB (g/dL)	13.56±0.58	13.49±0.26	13.46±0.65
Hematocrit, HCT (%)	42.01±1.66	41.85±0.80	41.73±1.60
Mean Corpuscular Volume, MCV (fL)	43.51±1.08	43.09±0.96	43.21±1.07
Mean Corpuscular Hemoglobin, MCH (pg)	14.04±0.23	13.88±0.23	13.94±0.30
Mean Corpuscular Hemoglobin Concentration, MCHC (g/dL)	32.27±0.75	32.21±0.33	32.25±0.58
Platelet, PLT (10^3/μL)	1723±215	1658±175	1723±266
Red Cell Distribution Width, RDW-SD (fL)	28.05±1.90	28.01±1.58	28.01±1.46
Red Cell Distribution Width, RDW-CV (%)	20.82±1.05	21.05±1.14	21.05±1.00
Platelet Distribution Width, PDW (fL)	6.96±0.19	6.92±0.18	6.87±0.08
Mean Platelet Volume, MPV (fL)	6.37±0.12	6.34±0.14	6.35±0.11
Platelet Large Cell Ratio, P-LCR (%)	3.42±0.59	3.31±0.60	3.27±0.51
Plateletcrit, PCT (%)	1.10±0.13	1.05±0.11	1.09±0.18
Neutrophils, NEUT (10^3/μL)	0.68±0.22	0.52±0.24	**0.50±0.16***
Lymphocytes, LYMPH (10^3/μL)	5.56±1.55	5.41±1.08	5.22±1.78
Monocytes, MONO (10^3/μL)	0.25±0.12	0.24±0.10	0.23±0.11
Eosinophils, EOS (10^3/μL)	0.05±0.03	0.05±0.04	0.07±0.03
Basophils, BASO (10^3/μL)	0.001±0.004	0.000±0.000	0.000±0.000
NEUT (%)	10.5±3.3	**8.0±2.2***	**8.5±1.6***
LYMPH (%)	84.7±4.5	87.4±3.2	86.6±1.8
MONO (%)	4.0±1.5	3.8±1.0	3.7±0.8
EOS (%)	0.74±0.34	0.77±0.46	**1.19±0.39***
BASO (%)	0.02±0.06	0.00±0.00	0.00±0.00
Reticulocytes, RET (10^6/μL)	0.3763±0.0538	0.3661±0.0393	0.3784±0.0719
RET (%)	3.90±0.55	3.77±0.41	3.94±0.93

*: $p < 0.05$ (compared with the vehicle group; unpaired Student's t test).

Historical control data

NEUT (10^3/μL): 1.086±0.467

NEUT (%): 13.18±4.62

EOS (%): 1.46±0.40

VG, viral genome.

Supplementary Table S6 Comprehensive hematology parameters on Day 91

Parameters	Dose (VG/kg)		
	-	5 x 10^{11}	5 x 10^{12}
White Blood Cells, WBCs (10^3/μL)	7.92±2.03	**9.54±1.51***	8.09±2.22
Red Blood Cells, RBCs (10^6/μL)	9.76±0.26	9.80±0.26	9.92±0.27
Hemoglobin, HGB (g/dL)	13.77±0.40	13.91±0.40	**14.06±0.25***
Hematocrit, HCT (%)	42.61±1.18	43.37±1.27	**44.13±0.91***
Mean Corpuscular Volume, MCV (fL)	43.63±0.79	**44.27±0.68***	**44.49±0.68***
Mean Corpuscular Hemoglobin, MCH (pg)	14.10±0.22	14.20±0.20	14.17±0.20
Mean Corpuscular Hemoglobin Concentration, MCHC (g/dL)	32.31±0.51	32.08±0.41	**31.86±0.33***
Platelet, PLT (10^3/μL)	1746±128	1734±175	1693±100
Red Cell Distribution Width, RDW-SD (fL)	27.89±1.02	27.89±0.76	28.25±0.78
Red Cell Distribution Width, RDW-CV (%)	20.88±0.67	20.83±0.53	21.16±0.54
Platelet Distribution Width, PDW (fL)	7.00±0.23	7.02±0.19	7.05±0.12
Mean Platelet Volume, MPV (fL)	6.38±0.15	6.41±0.10	6.38±0.09
Platelet Large Cell Ratio, P-LCR (%)	3.51±0.58	3.78±0.53	3.65±0.42
Plateletcrit, PCT (%)	1.13±0.08	1.11±0.11	1.08±0.06
Neutrophils, NEUT (10^3/μL)	0.83±0.32	0.82±0.25	0.66±0.16
Lymphocytes, LYMPH (10^3/μL)	6.63±1.69	**8.14±1.33***	6.95±2.00
Monocytes, MONO (10^3/μL)	0.35±0.11	**0.44±0.09***	0.37±0.11
Eosinophils, EOS (10^3/μL)	0.11±0.06	0.13±0.07	0.11±0.05
Basophils, BASO (10^3/μL)	0.002±0.004	0.004±0.005	0.003±0.005
NEUT (%)	10.4±3.3	8.7±2.5	8.4±1.9
LYMPH (%)	83.9±3.7	85.3±3.0	85.6±2.5
MONO (%)	4.4±0.8	4.6±0.6	4.6±0.7
EOS (%)	1.27±0.52	1.33±0.57	1.37±0.45
BASO (%)	0.02±0.04	0.04±0.05	0.03±0.05
Reticulocytes, RET (10^6/μL)	0.4082±0.0368	0.3979±0.0327	0.4126±0.0492
RET (%)	4.19±0.41	4.07±0.37	4.16±0.47

*: $p < 0.05$ (compared with the vehicle group; unpaired Student's t test).

Historical control data

WBCs (10^3/μL): 8.170±1.018

HGB (g/dL): 12.40±0.83

HCT (%): 39.56±3.30

MCV (fL): 46.16±2.90

MCHC (g/dL): 31.40±1.37

LYMPH (10^3/μL): 6.592±0.852
MONO (10^3/μL): 0.372±0.134
VG, viral genome.

Supplementary Table S7 Comprehensive biochemistry parameters on Day 2

Parameters	Dose (VG/kg)		
	-	5 x 10^{11}	5 x 10^{12}
Albumin, ALB (g/dL)	3.0±0.8	3.1±0.2	3.3±0.1
Total Protein, TP (g/dL)	5.7±0.3	5.6±0.8	5.9±0.2
Aspartate Aminotransferase, AST (U/L)	118±65	100±33	97±39
Alanine Aminotransferase, ALT (U/L)	42±26	40±27	39±19
Glucose, GLU (mg/dL)	267±53	245±73	302±54
Alkaline Phosphatase, ALP (U/L)	72±18	67±19	69±11
r-Glutamyl Transferase, r-GT (U/L)	1.3±0.5	1.4±0.5	1.6±0.5
Lactate Dehydrogenase, LDH (U/L)	344±180	284±143	298±69
Blood Urea Nitrogen, BUN (mg/dL)	30.4±5.0	31.7±14.0	31.6±4.7
Creatinine, CRE (mg/dL)	<0.2	<0.2	<0.2
Uric Acid, UA (mg/dL)	2.4±0.8	2.5±1.0	3.1±1.1
Direct Bilirubin, D-BIL (mg/dL)	0.04±0.01	0.04±0.02	0.05±0.02
Total Bilirubin, T-BIL (mg/dL)	0.08±0.03	0.09±0.03	0.08±0.05
Phosphorus, P (mg/dL)	7.9±2.0	6.7±2.3	8.0±1.3
Calcium, Ca (mg/dL)	8.4±1.5	8.1±2.7	**9.4±0.3***
Total Cholesterol, T-CHO (mg/dL)	81±18	75±28	88±11
Triglycerides, TG (mg/dL)	59±18	73±42	53±19
Creatine Phosphokinase, CPK (U/L)	437±401	**185±163***	**146±121***
Magnesium, Mg (mg/dL)	3.39±0.48	3.31±1.05	**3.72±0.18***
Sodium, Na (mEq/L)	150.9±2.2	151.4±2.6	151.0±2.1
Potassium, K (mEq/L)	4.43±0.75	4.40±0.45	4.51±0.23
Chloride, Cl (mEq/L)	114.5±2.2	115.3±2.2	114.7±2.0
Albumin/Globumin (A/G ratio)	1.16±0.32	1.25±0.06	0.57±2.30

*: $p < 0.05$ (compared with the vehicle group; unpaired Student's t test).

Historical control data

Ca (mg/dL): no reference

CPK (U/L): no reference

Mg (mg/dL): 2.814±0.298

VG, viral genome.

Supplementary Table S8 Comprehensive biochemistry parameters on Day 15

Parameters	Dose (VG/kg)		
	-	5 x 10^{11}	5 x 10^{12}
Albumin, ALB (g/dL)	2.7±0.1	2.8±0.1	2.8±0.1
Total Protein, TP (g/dL)	5.0±0.3	5.1±0.2	**5.2±0.1***
Aspartate Aminotransferase, AST (U/L)	164±88	159±66	161±67
Alanine Aminotransferase, ALT (U/L)	109±90	104±69	117±66
Glucose, GLU (mg/dL)	272±99	292±32	304±42
Alkaline Phosphatase, ALP (U/L)	86±31	95±10	97±11
r-Glutamyl Transferase, r-GT (U/L)	1.0±0.0	1.0±0.0	1.0±0.0
Lactate Dehydrogenase, LDH (U/L)	432±256	391±156	412±122
Blood Urea Nitrogen, BUN (mg/dL)	28.8±11.9	31.3±5.5	30.4±3.5
Creatinine, CRE (mg/dL)	0.22±0.02	0.22±0.02	0.22±0.01
Uric Acid, UA (mg/dL)	2.5±1.2	2.3±0.4	2.4±0.3
Direct Bilirubin, D-BIL (mg/dL)	0.03±0.01	0.03±0.01	0.03±0.01
Total Bilirubin, T-BIL (mg/dL)	0.06±0.02	<0.04	<0.04
Phosphorus, P (mg/dL)	7.2±2.6	**9.9±1.9***	**9.6±1.1***
Calcium, Ca (mg/dL)	8.1±2.9	9.2±0.3	9.3±0.2
Total Cholesterol, T-CHO (mg/dL)	83±29	99±8	97±12
Triglycerides, TG (mg/dL)	84±39	70±24	72±31
Creatine Phosphokinase, CPK (U/L)	1195±791	982±429	995±405
Magnesium, Mg (mg/dL)	3.19±1.02	3.67±0.22	3.48±0.26
Sodium, Na (mEq/L)	151.7±1.1	152.3±1.1	**152.9±1.2***
Potassium, K (mEq/L)	4.66±0.72	4.87±0.39	4.91±0.27
Chloride, Cl (mEq/L)	117.7±1.7	117.9±1.6	118.1±1.0
Albumin/Globumin (A/G ratio)	1.18±0.09	1.15±0.07	1.18±0.06

*: $p< 0.05$ (compared with the vehicle group; unpaired Student's t test).

Historical control data

TP (g/dL): 5.64±0.19

P (mg/dL): 10.30±2.04

Na (mEq/L): no reference

VG, viral genome.

Supplementary Table S9 Comprehensive biochemistry parameters on Day 29

Parameters	Dose (VG/kg)		
	-	5 x 10^{11}	5 x 10^{12}
Albumin, ALB (g/dL)	2.6±0.1	2.7±0.1	2.7±0.1
Total Protein, TP (g/dL)	4.7±0.7	5.0±0.2	5.0±0.1
Aspartate Aminotransferase, AST (U/L)	118±90	77±40	84±40
Alanine Aminotransferase, ALT (U/L)	57±49	52±41	69±49
Glucose, GLU (mg/dL)	267±81	292±44	294±41
Alkaline Phosphatase, ALP (U/L)	80±21	81±8	79±5
r-Glutamyl Transferase, r-GT (U/L)	1.0±0.0	1.0±0.0	1.0±0.0
Lactate Dehydrogenase, LDH (U/L)	311±182	248±121	303±159
Blood Urea Nitrogen, BUN (mg/dL)	34.5±13.5	31.4±4.5	35.2±7.4
Creatinine, CRE (mg/dL)	0.55±0.48	<0.2	<0.2
Uric Acid, UA (mg/dL)	1.7±0.4	1.9±0.2	**2.1±0.2***
Direct Bilirubin, D-BIL (mg/dL)	0.03±0.02	0.03±0.02	0.03±0.01
Total Bilirubin, T-BIL (mg/dL)	0.11±0.13	0.06±0.05	0.06±0.03
Phosphorus, P (mg/dL)	8.0±2.0	**9.4±1.2***	**9.7±1.3***
Calcium, Ca (mg/dL)	8.4±2.3	9.2±0.2	9.1±0.2
Total Cholesterol, T-CHO (mg/dL)	91±11	93±12	94±10
Triglycerides, TG (mg/dL)	80±26	**59±20***	67±24
Creatine Phosphokinase, CPK (U/L)	845±1072	403±395	451±338
Magnesium, Mg (mg/dL)	3.12±0.36	3.32±0.09	**3.35±0.19***
Sodium, Na (mEq/L)	148.2±1.4	148.6±1.4	**149.5±1.1***
Potassium, K (mEq/L)	4.67±0.55	4.7.0±0.53	4.90±0.32
Chloride, Cl (mEq/L)	115.2±1.5	115.3±1.2	**116.4±1.2***
Albumin/Globumin (A/G ratio)	0.77±1.65	1.13±0.07	1.17±0.05

*: $p< 0.05$ (compared with the vehicle group; unpaired Student's t test).

Historical control data

UA (mg/dL): 1.52±0.45

P (mg/dL): 10.30±2.04

TG (mg/dL): 48.6±13.2

Mg (mg/dL): 2.814±0.298

Na (mEq/L): no reference

Cl (mEq/L): no reference

VG, viral genome.

Supplementary Table S10 Comprehensive biochemistry parameters on Day 91

Parameters	Dose (VG/kg)		
	-	5 x 10^{11}	5 x 10^{12}
Albumin, ALB (g/dL)	2.7±0.1	2.8±0.1	**2.9±0.1***
Total Protein, TP (g/dL)	5.4±1.1	5.2±0.1	5.5±0.2
Aspartate Aminotransferase, AST (U/L)	154±90	104±40	113±52
Alanine Aminotransferase, ALT (U/L)	66±47	55±39	77±58
Glucose, GLU (mg/dL)	296±52	322±41	307±59
Alkaline Phosphatase, ALP (U/L)	66±6	67±6	**72±6***
r-Glutamyl Transferase, r-GT (U/L)	1.0±0.0	1.0±0.0	1.1±0.4
Lactate Dehydrogenase, LDH (U/L)	336±159	272±118	333±219
Blood Urea Nitrogen, BUN (mg/dL)	33.2±5.4	31.7±4.3	29.3±4.2
Creatinine, CRE (mg/dL)	0.21±0.01	<0.2	0.23±0.02
Uric Acid, UA (mg/dL)	1.6±0.6	1.9±0.4	1.9±0.5
Direct Bilirubin, D-BIL (mg/dL)	0.02±0.01	0.02±0.00	0.03±0.03
Total Bilirubin, T-BIL (mg/dL)	0.04±0.01	<0.04	<0.04
Phosphorus, P (mg/dL)	7.9±1.5	8.2±1.1	9.1±1.8
Calcium, Ca (mg/dL)	9.5±2.3	9.0±0.2	9.3±0.3
Total Cholesterol, T-CHO (mg/dL)	101±8	107±9	**115±11***
Triglycerides, TG (mg/dL)	86±30	84±16	75±23
Creatine Phosphokinase, CPK (U/L)	1026±868	684±406	601±380
Magnesium, Mg (mg/dL)	3.16±0.75	3.34±0.14	3.43±0.31
Sodium, Na (mEq/L)	147.7±2.1	147.1±1.9	**149.4±1.9***
Potassium, K (mEq/L)	4.36±0.48	4.33±0.30	4.56±0.68
Chloride, Cl (mEq/L)	114.6±3.0	113.9±2.4	114.6±2.6
Albumin/Globumin (A/G ratio)	1.09±0.19	1.14±0.05	1.11±0.05

*: p< 0.05 (compared with the vehicle group; unpaired Student's t test).

Historical control data

ALB (g/dL): 3.46±0.25

ALP (U/L): 88.6±20.2

BUN (mg/dL): 28.74±3.02

T-CHO (mg/dL): 97.0±4.2

Na (mEq/L): no reference

VG, viral genome.

Supplementary Table S11 Comprehensive urine quantitative analysis parameters on Day 2

Items	Critical Values	Dose (VG/kg)		
		-	5x10^{11}	5x10^{12}
Number of Animals Examined		15	15	15
Weight	Mean±SD	2069.1±766.0	2085.4±605.1	2067.2±593.9
	<1000 mg	0	1	0
	1000-2000 mg	7	7	6
	2001-3000 mg	7	6	9
	>3000 mg	1	1	0
Color	Yellow	14	15	15
	Colorless	1	0	0
	Red	0	0	0
	Light Orange	0	0	0
	Light Yellow	0	0	0
	Dark Yellow	0	0	0
	Orange	0	0	0
Odor	Normal	15	15	15
Turbidity	Clear	15	15	15
Glucose (Glu)	Normal	15	15	15
	Trace	0	0	0
Protein (PRO)	1+	12	11	13
	2+	3	4	2
	3+	0	0	0
Bilirubin (Bil)	Negative	15	14	14
	1+	0	1	1
Urobilinogen (URO)	Normal	4	2	3
	1+	11	11	11
	2+	0	2	1
pH Value	6.0-6.5	5	3	5
	7.0-7.5	10	11	10
	8.0-8.5	0	1	0
Specific gravity (SG)	1.010~1.015	1	0	2
	1.020~1.025	6	9	7
	>1.030	8	6	6
Blood (BLD)	Negative	15	15	15
	Trace	0	0	0
	1+	0	0	0
Ketones (KET)	Negative	0	2	1
	Trace	14	8	13
	1+	1	5	1
Nitrite (NIT)	Negative	15	15	15
	1+	0	0	0
Leukocyte Esterase (Leu)(Leu/μL)	Negative	2	2	1
	25-50	12	11	14
	51-75	1	2	0

VG, viral genome.

Supplementary Table S12 Comprehensive urine quantitative analysis parameters on Day 15

Items	Critical Values	Dose (VG/kg)		
		-	$5x10^{11}$	$5x10^{12}$
Number of Animals Examined		15	15	15
Weight	Mean±SD	1693.8±607.5	1566.1±442.2	1733.4±506.3
	<1000 mg	1	2	1
	1000-2000 mg	9	11	9
	2001-3000 mg	5	2	5
	>3000 mg	0	0	0
Color	Yellow	14	13	15
	Colorless	0	0	0
	Red	1	1	0
	Light Orange	0	1	0
	Light Yellow	0	0	0
	Dark Yellow	0	0	0
	Orange	0	0	0
Odor	Normal	15	15	15
Turbidity	Clear	15	15	15
Glucose (Glu)	Normal	15	15	15
	Trace	0	0	0
Protein (PRO)	1+	7	9	10
	2+	7	6	5
	3+	1	0	0
Bilirubin (Bil)	Negative	15	15	15
	1+	0	0	0
Urobilinogen (URO)	Normal	3	2	4
	1+	11	11	11
	2+	1	2	0
pH Value	6.0-6.5	1	0	3
	7.0-7.5	12	10	10
	8.0-8.5	2	5	2
Specific Gravity (SG)	1.010~1.015	0	0	1
	1.020~1.025	9	7	6
	>1.030	6	8	8
Blood (BLD)	Negative	15	14	15
	Trace	0	1	0
	1+	0	0	0
Ketones (KET)	Negative	2	0	0
	Trace	8	10	13
	1+	5	5	2
Nitrite (NIT)	Negative	15	15	15
	1+	0	0	0
Leukocyte Esterase (Leu)(Leu/µL)	Negative	5	1	2
	25-50	6	11	13
	51-75	4	3	0

VG, viral genome.

Supplementary Table S13 Comprehensive urine quantitative analysis parameters on Day 29

Items	Critical Values	Dose (VG/kg)		
		-	5x10^{11}	5x10^{12}
Number of Animals Examined		15	15	15
Weight	Mean±SD	1489.8±568.0	1557.4±354.9	1295.9±515.9
	<1000 mg	3	0	5
	1000-2000 mg	10	14	8
	2001-3000 mg	2	1	2
	>3000 mg	0	0	0
Color	Yellow	11	14	14
	Colorless	1	0	0
	Red	0	0	0
	Light Orange	2	1	0
	Light Yellow	1	0	0
	Dark Yellow	0	0	0
	Orange	0	0	1
Odor	Normal	15	15	15
Turbidity	Clear	15	15	15
Glucose (Glu)	Normal	15	15	14
	Trace	0	0	1
Protein (PRO)	1+	8	9	8
	2+	7	6	7
	3+	0	0	0
Bilirubin (Bil)	Negative	15	15	15
	1+	0	0	0
Urobilinogen (URO)	Normal	7	4	4
	1+	8	11	10
	2+	0	0	1
pH Value	6.0-6.5	2	1	4
	7.0-7.5	12	11	7
	8.0-8.5	1	3	4
Specific Gravity (SG)	1.010~1.015	0	1	0
	1.020~1.025	11	7	11
	>1.030	4	7	4
Blood (BLD)	Negative	14	15	15
	Trace	0	0	0
	1+	1	0	0
Ketones (KET)	Negative	0	0	0
	Trace	11	9	5
	1+	4	6	10
Nitrite (NIT)	Negative	11	15	11
	1+	4	0	4
Leukocyte Esterase (Leu)(Leu/μL)	Negative	9	3	2
	25-50	6	12	11
	51-75	0	0	2

VG, viral genome.

Supplementary Table S14 Comprehensive urine quantitative analysis parameters on Day 91

Items	Critical Values	Dose (VG/kg)		
		-	$5x10^{11}$	$5x10^{12}$
Number of Animals Examined		15	15	15
Weight	Mean±SD	1647.3±889.3	1355.7±423.1	1186.8±520.9
	<1000 mg	3	5	5
	1000-2000 mg	10	10	10
	2001-3000 mg	1	0	0
	>3000 mg	1	0	0
Color	Yellow	11	13	11
	Colorless	2	0	2
	Red	0	0	0
	Light Orange	1	0	0
	Light Yellow	1	2	1
	Dark Yellow	0	0	1
	Orange	0	0	0
Odor	Normal	15	15	15
Turbidity	Clear	15	15	15
Glucose (Glu)	Normal	15	14	15
	Trace	0	1	0
Protein (PRO)	1+	8	9	10
	2+	7	6	5
	3+	0	0	0
Bilirubin (Bil)	Negative	15	15	15
	1+	0	0	0
Urobilinogen (URO)	Normal	7	4	9
	1+	8	11	6
	2+	0	0	0
pH Value	6.0-6.5	8	6	3
	7.0-7.5	7	9	10
	8.0-8.5	0	0	2
Specific Gravity (SG)	1.010~1.015	0	0	5
	1.020~1.025	9	7	9
	>1.030	6	8	1
Blood (BLD)	Negative	15	14	14
	Trace	0	1	0
	1+	0	0	1
Ketones (KET)	Negative	2	1	4
	Trace	9	8	7
	1+	4	6	4
Nitrite (NIT)	Negative	15	15	15
	1+	0	0	0
Leukocyte Esterase (Leu)(Leu/μL)	Negative	4	2	4
	25-50	6	12	8
	51-75	5	1	2
	>75	0	0	1

VG, viral genome.
