# Supplementary figures and images for: Preclinical Evaluation of the Systemic Safety, Efficacy, and Biodistribution of a Recombinant AAV8 Vector Expressing FIX-TripleL in Hemophilia B Mice: Implications for Human Gene Therapy
Source: Int J Mol Sci. 2025 Jun 24;26(13):6073. doi: 10.3390/ijms26136073 (PMC12250290; doi:10.3390/ijms26136073)

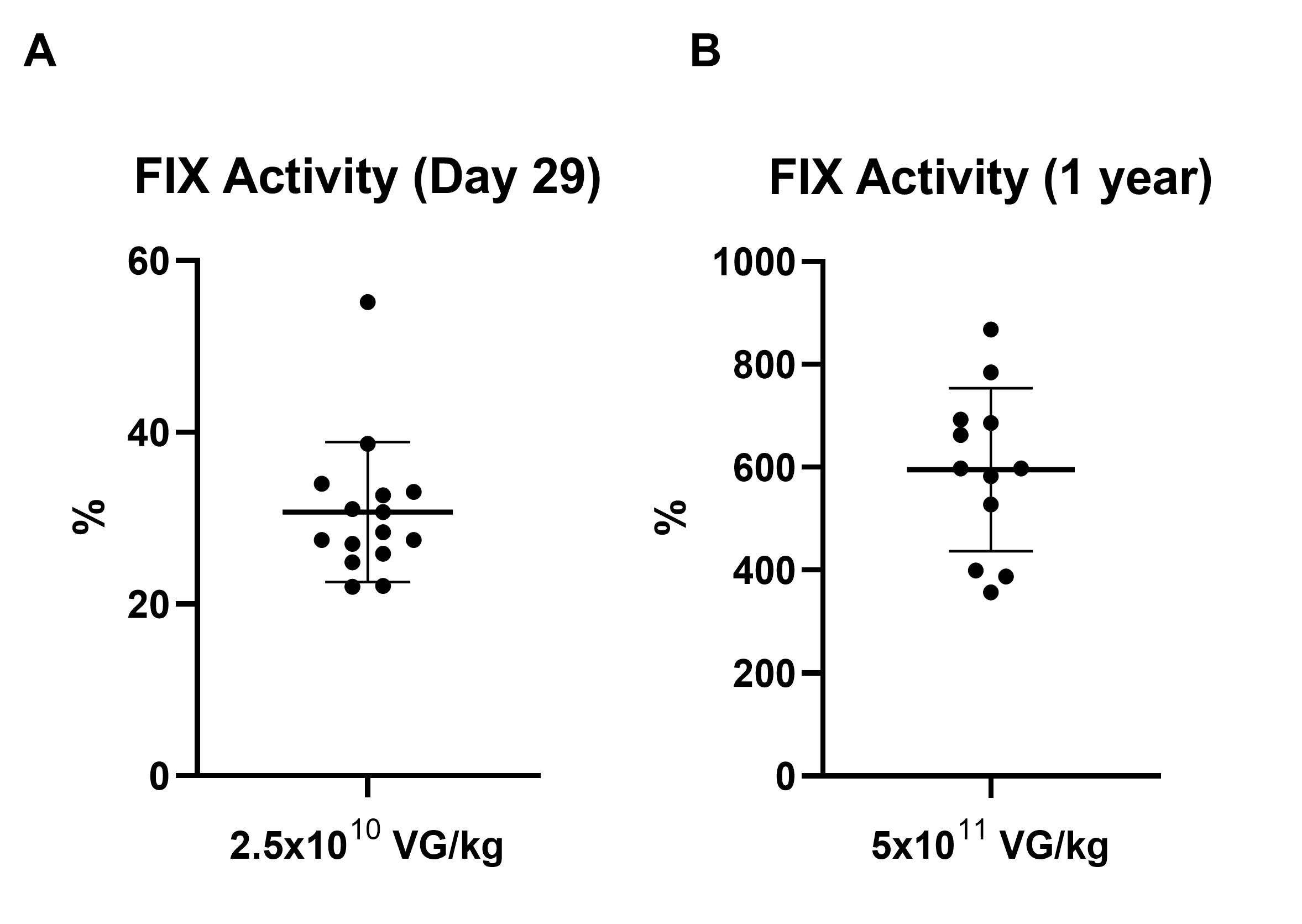

Supplement: Supplementary file 1 [file ijms-26-06073-s001.zip › Sup Fig S1 FIX activity.tif]

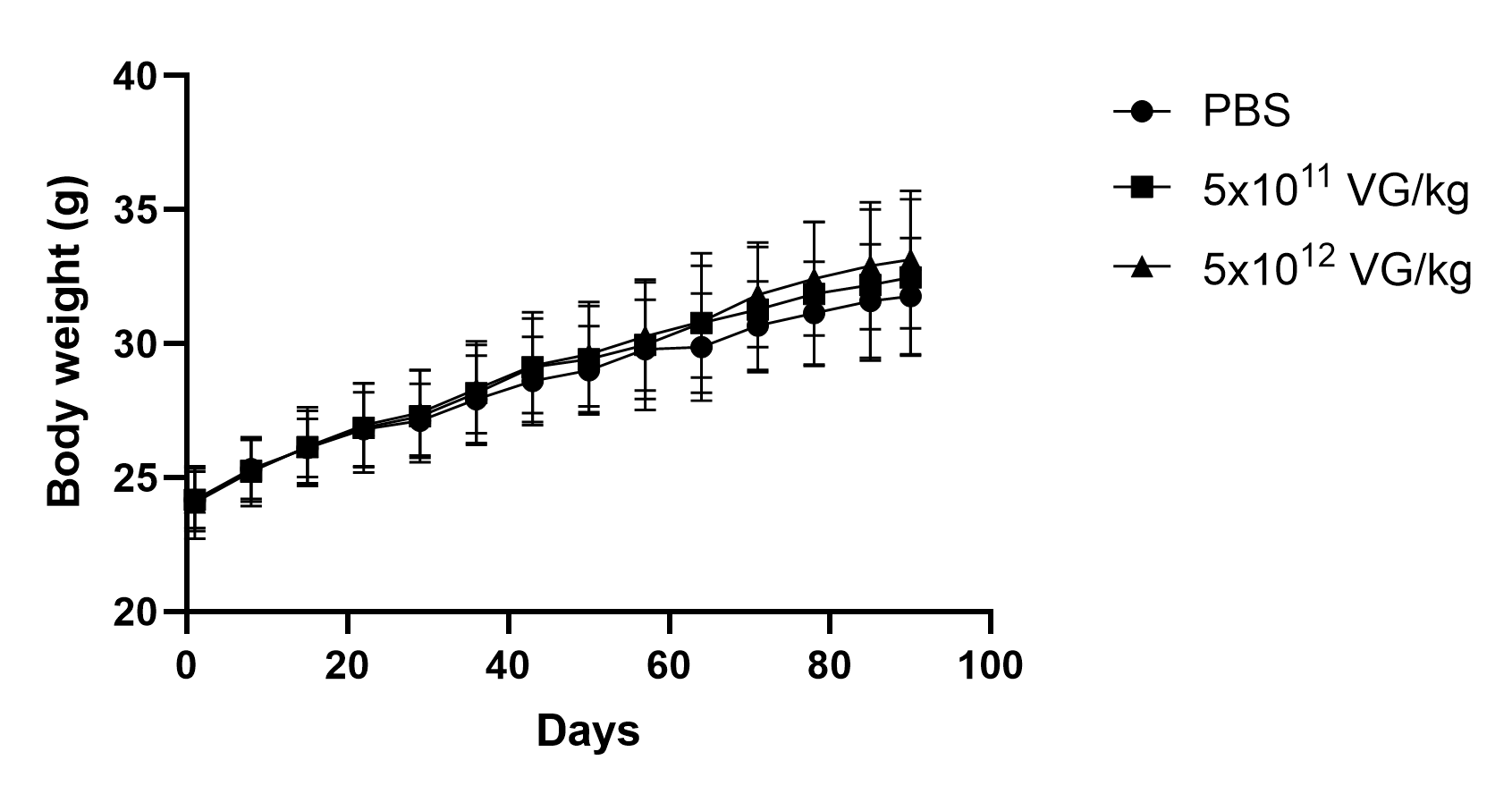

Supplement: Supplementary file 1 [file ijms-26-06073-s001.zip › Sup Fig S2 B.W.tif]

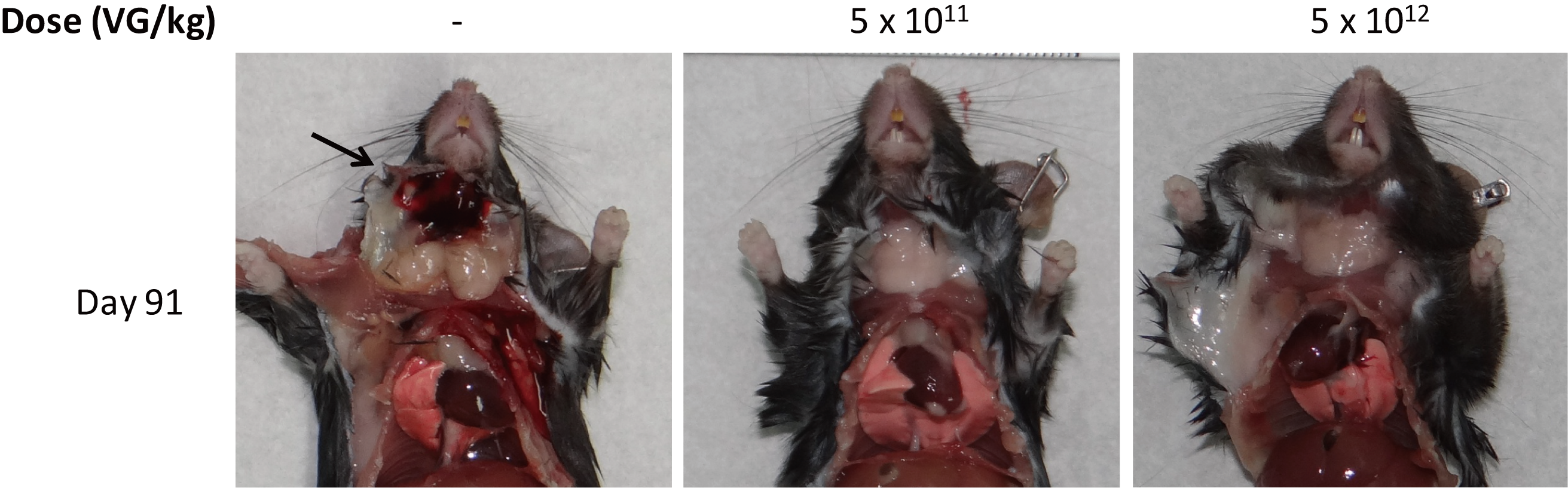

Supplement: Supplementary file 1 [file ijms-26-06073-s001.zip › Sup Fig S3.tif]
